# Clinical Presentation and Management of Acute Exacerbation of Asthma During Pregnancy at a Tertiary Care Hospital

**DOI:** 10.7759/cureus.97309

**Published:** 2025-11-20

**Authors:** Hafiz Syed Ahmad Hassan, Rabeea Komal, Abdurrab Khan, Mehr Ali Khan

**Affiliations:** 1 Department of Medicine, Services Institute of Medical Sciences, Lahore, PAK; 2 Department of Obstetrics and Gynaecology, Jinnah International Hospital Abbottabad/Women Medical College, Abbottabad, PAK; 3 Department of Medicine and Pulmonology, Jinnah International Hospital Abbottabad/Women Medical College, Abbottabad, PAK; 4 Department of General Medicine and Pulmonology, Chest Clinic, Shafiq Medical Centre, Abbottabad, PAK; 5 Department of Pulmonology, Ayub Teaching Hospital, Abbottabad, PAK; 6 Department of Pulmonary Medicine, Tallaght University Hospital, Dublin, IRL

**Keywords:** acute asthma exacerbation, asthma, maternal health, pregnancy, pregnancy outcome, pulmonary function tests

## Abstract

Introduction

Asthma is a chronic airway disorder posing significant risks during pregnancy. Acute exacerbations compromise maternal oxygenation and increase adverse perinatal outcomes, yet data from low- and middle-income countries are limited.

Objective

The objective of this study was to evaluate the clinical presentation, management strategies, and maternal-fetal outcomes of acute asthma exacerbations during pregnancy in a tertiary care hospital, and to highlight differences from patterns reported in high-income settings.

Methodology

This descriptive observational study was conducted at Ayub Teaching Hospital, Abbottabad, Pakistan, from March 2021 to March 2022. A total of 106 pregnant women with acute exacerbation were included. Clinical assessment covered symptoms, peak expiratory flow rate (PEFR), oxygen saturation, and parity. Management followed hospital protocols with short-acting beta-agonists (SABA), systemic corticosteroids, oxygen supplementation, and, when required, intravenous magnesium sulfate or mechanical ventilation. Maternal and fetal outcomes were recorded. Data were analyzed using IBM SPSS Statistics for Windows, version 25 (IBM Corp., Armonk, New York, United States); chi-square and Kruskal-Wallis tests assessed variables, and multivariate logistic regression identified predictors (p<0.05).

Results

Mild and moderate exacerbations accounted for 42 (39.6%) and 46 (43.4%) cases, while 18 (17.0%) were severe. Primigravida women comprised 42.5% (n=45) of the cohort. PEFR and oxygen saturation declined with severity (p<0.001). All patients received SABA; corticosteroids and oxygen were more common in moderate and severe cases. IV magnesium sulfate (n=11/18, 61.1%) and mechanical ventilation (n=4/18, 22.2%) were mainly required in severe cases. Maternal complications, including hypoxemia (n=9/18, 50.0%) and ICU admission (n=12/18, 66.7%), rose with severity (p<0.001). Fetal complications were higher in severe cases, but were not statistically significant.

Conclusion

Acute asthma exacerbations in pregnancy are mainly mild to moderate. Severe cases carry substantial maternal risk, underscoring early recognition, guideline-based management, and multidisciplinary care.

## Introduction

Asthma is a chronic inflammatory illness of the airways that causes coughing, dyspnea, wheezing, and tightness in the chest [1]. Globally, it affects approximately 300 million individuals, with a substantial proportion of cases occurring in women of reproductive age [2]. During pregnancy, certain specific physiological and immunological changes that may affect the progression of the asthma condition occur, complicating the treatment process [3]. These changes include increased oxygen consumption, increased position of the diaphragm, decreased functional residual volume, and hormonal changes that can lead to a change in airways reactivity [4].

Pregnancy during an acute exacerbation of asthma is a risk to both mother and child [5]. Potential maternal complications could be hypoxemia, respiratory failure, and preterm labor, and potential fetal complications could be intrauterine growth restriction, low birth weight, preterm birth, and, in the worst-case scenario, perinatal mortality. According to studies, poorly controlled asthma is linked to higher rates of cesarean delivery, preeclampsia, and placental complications [6]. On the contrary, these negative consequences can be alleviated by optimal management of asthma exacerbations, and it is necessary to recognize and intervene as soon as possible [7].

Pregnant women may have a similar clinical picture of acute asthma exacerbation as the general population, with such symptoms as severe dyspnea, wheezing, cough, and chest tightness [8]. However, pregnancy can confound the severity of airflow obstruction with physiological changes, making clinical evaluation difficult. For the assessment of the severity of exacerbations, pulmonary function testing (especially peak expiratory flow rate, peak expiratory flow rate (PEFR), and spirometry) and oxygen saturation analysis are the necessary tools. Also, cases of severe hypoxemia or hypercapnia may need lab tests and blood analysis of arterial blood to determine the degree of hypoxemia or hypercapnia [9].

Pregnancy requires careful consideration of the balance between maternal stabilization and fetal safety during the management of acute asthma exacerbations [10]. Standard treatment includes inhaled short-acting beta-agonists, systemic corticosteroids, supplemental oxygen, and, in severe cases, intravenous magnesium sulfate or mechanical ventilation [11]. Close monitoring in a tertiary care unit is essential to promptly identify deterioration and implement timely interventions [12]. Furthermore, patient education on adherence to maintenance therapy, avoidance of triggers, and early recognition of worsening symptoms is vital for preventing severe exacerbations [13]. Multidisciplinary care involving obstetricians, pulmonologists, and critical care specialists is crucial for optimizing both maternal and fetal outcomes [14].

Despite well-established international guidelines on asthma management in pregnancy [1,8,10,11,13-15], data on the clinical presentation, severity, and outcomes of acute exacerbations remain scarce in low- and middle-income countries. In contrast, studies conducted in well-resourced healthcare systems, including Canada [5], the Netherlands [14], and Sweden [16], have demonstrated better asthma control and improved maternal-fetal outcomes attributed to structured antenatal follow-up, early diagnosis, and adherence to maintenance therapy. However, such systematic approaches are often underutilized in resource-limited healthcare systems. Therefore, this study aimed to evaluate the clinical presentation, management strategies, and maternal and fetal outcomes of acute asthma exacerbations during pregnancy in a tertiary care hospital, addressing the existing gaps in regional evidence and emphasizing the need for context-specific management strategies.

## Materials and methods

Study design and setting

This descriptive observational study was carried out at the Department of Obstetrics and Gynecology, Ayub Teaching Hospital, Abbottabad, Pakistan. The study spanned a duration of 12 months, from March 2021 to March 2022, and included pregnant women presenting with acute exacerbation of asthma during this period.

Sample size calculation

The minimum sample size was calculated using the single-population proportion formula: $n_0 = \frac{Z_{\alpha/2}^{2} \, p \, (1-p)}{d^{2}}$, where Z=1.96Z (95% confidence), P=0.477 (prevalence of acute asthma exacerbation during pregnancy) [17], and d is the desired margin of error. A sample size of 96 was obtained using d=0.10 (10% margin of error). A 10% contingency was included to permit incomplete records, which resulted in a final target sample of 106 participants. A narrower margin of error (d=0.05) would have necessitated a much larger sample (n=383), which would not have been possible under the available resources and single-centre study design; thus, a margin of 10% was selected and is reported here with acknowledgement of the resulting lower precision.

Inclusion and exclusion criteria

Pregnant women of any gestational age who had previously been diagnosed with asthma or who were diagnosed during pregnancy and exhibited symptoms suggestive of an acute asthma attack were included in the study. Individuals with interstitial lung disease or chronic obstructive pulmonary disease (COPD) were excluded. Additionally, cases where acute exacerbations were triggered by respiratory infections unrelated to asthma were excluded. Pregnant women who declined to provide informed consent were also excluded from the study.

Data collection

All enrolled patients underwent a detailed history and thorough physical examination at the time of presentation. The clinical parameters documented included age, parity, gestational age, severity of asthma exacerbation, and associated comorbidities. The degree of asthma severity was categorized according to the Global Initiative for Asthma (GINA) criteria into mild, moderate, and severe attacks [15]. A mild exacerbation was defined by the presence of dyspnea only on exertion, with the patient able to speak in full sentences, a pulse rate of less than 100 beats per minute, a respiratory rate below 25 breaths per minute, and a PEFR of 70% or more of the predicted value or personal best.

A moderate exacerbation was characterized by dyspnea interfering with usual activity, speech limited to phrases, a pulse rate of 100-120 beats per minute, a respiratory rate of 25-30 breaths per minute, and a PEFR between 40% and 69% of the predicted value. A severe exacerbation was defined by dyspnea at rest, speech limited to single words, use of accessory respiratory muscles, a pulse rate exceeding 120 beats per minute, a respiratory rate greater than 30 breaths per minute, oxygen saturation below 90%, and a PEFR less than 40% of the predicted value.

Pulmonary function was evaluated primarily by measuring the PEFR [18], and spirometry was performed when clinically indicated. Oxygen saturation was continuously monitored using pulse oximetry. Laboratory investigations, including complete blood count, arterial blood gases, and serum electrolytes, were carried out as required. The fetal condition was assessed through obstetric ultrasound and non-stress testing, where necessary to ensure continuous maternal and fetal monitoring.

Information regarding the patients’ asthma control status prior to the acute attack was obtained from medical records and patient interviews. Details about maintenance therapy-such as the use of inhaled corticosteroids, long-acting β₂-agonists, anticholinergic agents, and adherence to controller medication-were recorded when available. Data on prescribed medications and follow-up plans after discharge were also collected to evaluate continuity of asthma care.

Management protocol

Patients were managed according to standard hospital protocols and GINA-based guidelines for acute asthma exacerbation in pregnancy. This included administration of inhaled short-acting beta-agonists (SABA), systemic corticosteroids, supplemental oxygen, and, in severe cases, intravenous magnesium sulfate or mechanical ventilation [19]. All patients were closely monitored for maternal and fetal complications throughout hospitalization.

Data analysis

The analysis and entry of data were done in IBM SPSS Statistics for Windows, version 25 (IBM Corp., Armonk, New York, United States). Comparisons of pre-attack medication use and post-discharge treatment plans were also analyzed descriptively. Continuous variables were expressed as mean ± SD or median (interquartile range (IQR)) as appropriate. For continuous variables compared across severity groups, the Kruskal-Wallis test was used to account for non-normal distribution. Statistical significance was defined as a p-value <0.05. In order to find independent determinants of maternal and fetal outcomes, multivariate logistic regression analysis was used.

Ethical consideration

The study was approved by the Institutional Review Board of Ayub Teaching Hospital (approval number: ATH/EC-1/21(7), dated February 16, 2021), and each participant gave their informed consent.

## Results

A total of 106 pregnant women presenting with acute exacerbation of asthma were included in the study. The majority of exacerbations were classified as mild (n=42, 39.6%) and moderate (n=46, 43.4%), while 18 (17.0%) patients had severe exacerbations according to GINA guidelines [15]. As shown in Table 1, the mean age of participants was 28.6 ± 5.2 years, and the mean gestational age at presentation was 27.8 ± 6.4 weeks. Primigravida women comprised 42.5% (n=45) of the cohort. The mean PEFR decreased significantly with increasing severity (298.2 ± 32.5, 262.4 ± 28.6, and 205.6 ± 24.8 L/minute for mild, moderate, and severe exacerbations, respectively), and oxygen saturation also declined (96.0 ± 2.0%, 93.0 ± 3.0%, and 88.0 ± 4.0%, respectively; p<0.001). Age, gestational age, and parity did not differ significantly across severity groups. In terms of parity distribution, primigravida women accounted for 17 (40.5%), 20 (43.5%), and eight (44.4%) mild, moderate, and severe cases, respectively, while multigravida women comprised 25 (59.5%), 26 (56.5%), and 10 (55.6%) in the respective groups.

**Table 1:** Baseline demographic and clinical characteristics by severity Continuous variables expressed as mean ± SD; compared by Kruskal–Wallis test. Categorical variables compared by χ² test. *p<0.05 is significant. “Severity” refers to asthma exacerbation severity PEFR: peak expiratory flow rate

Variable		Mild (n=42)	Moderate (n=46)	Severe (n=18)	Test statistic	p-value
Age (years), mean ± SD		28.3 ± 5.0	28.8 ± 5.3	29.1 ± 5.7	H = 0.68	0.71
Gestational age (weeks), mean ± SD		27.5 ± 6.2	27.8 ± 6.5	28.6 ± 6.6	H = 0.37	0.83
Median parity		1	1	1	H = 0.17	0.92
Parity (categorical), n (%)	Primigravida	17 (40.5%)	20 (43.5%)	8 (44.4%)	χ² = 0.26	0.88
	Multigravida	25 (59.5%)	26 (56.5%)	10 (55.6%)		
PEFR (L/min), mean ± SD		298.2 ± 32.5	262.4 ± 28.6	205.6 ± 24.8	H = 51.27	<0.001*
Oxygen saturation (%), mean ± SD		96.0 ± 2.0	93.0 ± 3.0	88.0 ± 4.0	H = 49.85	<0.001*

Laboratory and clinical findings corresponding to the severity of asthma exacerbation are summarized in Table 2. Overall, cough, chest tightness, and dyspnea were the predominant symptoms across all severity levels, with no statistically significant variation between groups (p > 0.05). This suggests that while these symptoms are common markers of exacerbation, they do not reliably distinguish the degree of severity on their own.

**Table 2:** Association of clinical symptoms and laboratory findings with severity of asthma exacerbation. Categorical variables were compared using the Chi-square (χ²) test. A p-value of <0.05 was considered statistically significant (*p < 0.05). The severity of asthma exacerbation was classified according to GINA criteria. Abnormal ABG findings were defined primarily as hypoxemia and respiratory acidosis. GINA: Global Initiative for Asthma; CBC: complete blood count; ABG: arterial blood gas

Parameter	Mild (n=42), n (%)	Moderate (n=46), n (%)	Severe (n=18), n (%)	χ² value	p-value
Dyspnea	18 (42.9%)	24 (52.2%)	12 (66.7%)	0.15	0.87
Chest Tightness	25 (59.5%)	28 (60.9%)	12 (66.7%)	0.15	0.82
Cough	30 (71.4%)	34 (73.9%)	14 (77.8%)	0.15	0.82
Leukocytosis (CBC)	6 (14.3%)	9 (19.6%)	5 (27.8%)	1.93	0.38
Abnormal ABG	5 (11.9%)	6 (13.0%)	7 (38.9%)	8.71	0.01*

In contrast, laboratory assessments revealed clearer trends. Leukocytosis was more frequent among patients with moderate and severe attacks, although this difference was not statistically significant. However, abnormalities in arterial blood gases-particularly hypoxemia and respiratory acidosis-were markedly higher in patients with severe exacerbations, showing a significant association with clinical severity (p = 0.01). These findings underscore the importance of integrating objective laboratory parameters, such as ABG analysis, alongside symptom assessment for accurate evaluation of exacerbation severity.

The management strategies administered to patients with acute asthma exacerbation are summarized in Table 3. All patients received SABA as first-line therapy. Systemic corticosteroids were administered in 34 (81.0%) mild, 44 (95.7%) moderate, and 14 (77.8%) severe cases (p=0.03). Oxygen supplementation was given to four (9.5%) mild, 12 (26.1%) moderate, and 12 (66.7%) severe cases (p<0.001). Intravenous magnesium sulfate and mechanical ventilation were predominantly required in the severe group: 11 (61.1%) and four (22.2%), respectively (p<0.001). ICU admission increased significantly with severity, occurring in one (2.4%) mild, nine (19.6%) moderate, and 12 (66.7%) severe cases (p<0.001). Median hospital length of stay also increased with severity: two (IQR 1-2), three (IQR 2-4), and six (IQR 4-8) days in mild, moderate, and severe cases, respectively (p<0.001).

**Table 3:** Management and interventions. Data given as n (%) unless otherwise indicated. χ² test for categorical variables; Kruskal–Wallis test for length of stay (in days). *p<0.05 (significant). SABA: short-acting beta-agonists; ICU: intensive care unit; IQR: interquartile range

Intervention	Mild (n=42)	Moderate (n=46)	Severe (n=18)	Test statistic	p-value
SABA	42 (100%)	46 (100%)	18 (100%)	—	—
Systemic corticosteroids	34 (81.0%)	44 (95.7%)	14 (77.8%)	χ² = 6.99	0.03*
Oxygen supplementation	4 (9.5%)	12 (26.1%)	12 (66.7%)	χ² = 22.35	<0.001*
IV Magnesium sulfate	0 (0%)	2 (4.3%)	11 (61.1%)	χ² = 46.77	<0.001*
Mechanical ventilation	0 (0%)	1 (2.2%)	4 (22.2%)	χ² = 21.08	<0.001*
ICU admission	1 (2.4%)	9 (19.6%)	12 (66.7%)	χ² = 37.29	<0.001*
Length of stay (days), median (IQR)	2 (1–2)	3 (2–4)	6 (4–8)	H = 40.61	<0.001*

Maternal complications increased with the severity of asthma exacerbation, as shown in Table 4*.* Maternal hypoxemia occurred in three (7.1%) mild, seven (15.2%) moderate, and nine (50.0%) severe cases (p<0.001). Preterm labor was reported in four (9.5%) mild, eight (17.4%) moderate, and four (22.2%) severe cases (p=0.034). Cesarean delivery rates did not differ significantly across severity groups: 13 (31.0%), 17 (37.0%), and seven (38.9%) in mild, moderate, and severe cases, respectively (p=0.72). The incidence of any maternal complication increased significantly with severity, occurring in six (14.3%) mild, 17 (37.0%) moderate, and 13 (72.2%) severe cases (p<0.001).

**Table 4:** Maternal complications by severity of asthma exacerbation χ² test used for categorical comparisons. *p<0.05 significant.

Complication	Mild (n=42), n (%)	Moderate (n=46), n (%)	Severe (n=18), n (%)	χ² value	p-value
Maternal hypoxemia	3 (7.1%)	7 (15.2%)	9 (50.0%)	18.62	<0.001*
Preterm labor	4 (9.5%)	8 (17.4%)	4 (22.2%)	6.79	0.034*
Cesarean delivery	13 (31.0%)	17 (37.0%)	7 (38.9%)	0.66	0.72
Any maternal complication	6 (14.3%)	17 (37.0%)	13 (72.2%)	19.43	<0.001*

As shown in Table 5, preterm birth occurred in five (11.9%) mild, nine (19.6%) moderate, and four (22.2%) severe cases (p=0.221). Low birth weight was reported in six (14.3%) mild, 10 (21.7%) moderate, and five (27.8%) severe cases (p=0.112). Intrauterine growth restriction (IUGR) occurred in three (7.1%) mild, four (8.7%) moderate, and two (11.1%) severe cases (p=0.82). Perinatal mortality was observed in zero (0%) mild, one (2.2%) moderate, and one (5.6%) severe cases (p=0.34). The incidence of any fetal complication increased with maternal severity, with nine (21.4%), 14 (30.4%), and eight (44.4%) in mild, moderate, and severe cases, respectively (p=0.15), but these differences did not reach statistical significance.

**Table 5:** Fetal/perinatal outcomes. Chi-square (χ²) test used. No fetal outcomes reached statistical significance at p<0.05.

Outcome	Mild (n=42), n (%)	Moderate (n=46), n (%)	Severe (n=18), n (%)	χ² value	p-value
Preterm birth	5 (11.9%)	9 (19.6%)	4 (22.2%)	3.02	0.221
Low birth weight	6 (14.3%)	10 (21.7%)	5 (27.8%)	4.39	0.112
Intrauterine growth restriction	3 (7.1%)	4 (8.7%)	2 (11.1%)	0.40	0.820
Perinatal mortality	0 (0%)	1 (2.2%)	1 (5.6%)	2.17	0.340
Any fetal complication	9 (21.4%)	14 (30.4%)	8 (44.4%)	3.79	0.150

To find independent predictors of fetal complications, multivariate logistic regression analysis was performed. As shown in Table 6, the severity of maternal asthma exacerbation showed a trend toward an increased risk of fetal complications (β = 0.679; OR 1.97; 95%CI 0.90-4.34; p=0.091), while systemic corticosteroid therapy demonstrated a protective trend (β = -1.602; OR 0.20; 95%CI 0.03-1.20; p=0.079). Maternal hypoxemia (β = -0.059; OR 0.94; 95%CI 0.32-2.77; p=0.915) and gestational age (β = -0.040; OR 0.96; 95%CI 0.92-1.00; p=0.078) were not significant predictors. However, none of the variables reached statistical significance at p<0.05.

**Table 6:** Multivariate logistic regression analysis of factors associated with fetal complications. Logistic regression was adjusted for maternal hypoxemia, steroid use, and gestational age. "Severity" refers to the category of asthma exacerbation severity.

Variable	β Coefficient	OR	95% CI	p-value
Severity of asthma exacerbation (ordinal)	0.679	1.97	0.90–4.34	0.091
Maternal hypoxemia	-0.059	0.94	0.32–2.77	0.915
Systemic corticosteroid therapy	-1.602	0.20	0.03–1.20	0.079
Gestational age (weeks)	-0.040	0.96	0.92–1.00	0.078

## Discussion

This study assessed the clinical presentation, management, and outcomes of acute asthma exacerbation during pregnancy in a tertiary care setting. Most patients had mild to moderate exacerbations, with severe cases comprising 18 (17.0%). Primigravida women accounted for 45 (42.5%), with a similar distribution across severity groups. Pulmonary function (PEFR) decreased with severity (mild 298.2 ± 32.5, moderate 262.4 ± 28.6, severe 205.6 ± 24.8 L/minute), and oxygen saturation declined in severe cases (mild 96.0 ± 2.0%, moderate 93.0 ± 3.0%, severe 88.0 ± 4.0%). All patients received SABA, while systemic corticosteroids, oxygen supplementation, IV magnesium sulfate, and mechanical ventilation were more common in severe cases. Maternal complications, including hypoxemia, ICU admission, and preterm labor, increased with severity. Fetal outcomes tended to worsen in severe maternal exacerbations, but differences were not statistically significant. Logistic regression indicated a protective trend of corticosteroid therapy on fetal complications, though it was not significant.

The overall prevalence of severe asthma exacerbations observed in this study aligns with prior reports, indicating that most pregnant women experience mild to moderate exacerbations, while a subset remains at high risk for severe complications [20]. The decline in PEFR and oxygen saturation with increasing severity corroborates the well-established correlation between pulmonary function and exacerbation intensity [21].

The management strategies employed in this cohort reflect guideline-recommended practices, with universal use of SABA and frequent use of systemic corticosteroids and supplemental oxygen in moderate to severe cases [22]. The selective use of IV magnesium sulfate and mechanical ventilation in the most severe patients is consistent with best-practice recommendations for acute management [23]. Length of hospital stay and ICU admission rates increased with severity, highlighting the burden of severe exacerbations on tertiary care resources [24].

Maternal complications observed, particularly hypoxemia, preterm labor, and composite adverse events, were more prevalent in patients with severe exacerbations [16]. Cesarean delivery rates did not vary significantly with severity, suggesting that delivery mode may be influenced by multiple obstetric factors beyond asthma control [25]. Fetal outcomes, including preterm birth, low birth weight, and intrauterine growth restriction, were higher in severe maternal exacerbations but not statistically significant, likely reflecting the sample size or effectiveness of timely intervention [26]. Trends toward improved fetal outcomes with systemic corticosteroid therapy support the protective effect of appropriate maternal management [27].

Limitations and future recommendations

Several limitations should be acknowledged. First, this was a single-center study, limiting generalizability to other settings. Second, although adequate for descriptive analysis, the sample size may have been insufficient to detect statistically significant differences in fetal outcomes. Third, hospital-based recruitment may have excluded patients with mild exacerbations managed in outpatient settings. Fourth, pre-attack maintenance therapy varied among participants, and data on the use of inhaled corticosteroids, long-acting β₂-agonists, and anticholinergic agents prior to exacerbation were not consistently documented. Fifth, information on post-discharge medication adherence and follow-up care was limited. Lastly, the observational design restricts causal inference, and unmeasured confounders may have influenced outcomes.

Future research should include multicenter studies with larger sample sizes to better assess the impact of acute exacerbations on maternal and fetal outcomes. Prospective studies incorporating standardized pulmonary function assessments, detailed documentation of pre- and post-exacerbation medication use, and long-term neonatal follow-up would enhance understanding of the consequences of asthma exacerbations during pregnancy. Interventions targeting early identification, patient education, and adherence to maintenance therapy should be evaluated for their effectiveness in reducing severe exacerbations and improving maternal-fetal outcomes. Additionally, the use of brief patient-reported measures could be explored in busy clinical settings to efficiently monitor maternal well-being and predict adverse outcomes.

## Conclusions

Acute exacerbation of asthma during pregnancy predominantly presents as mild to moderate; however, severe cases carry a significant risk for maternal complications, including hypoxemia, ICU admission, and the need for mechanical ventilation. Pulmonary function, as measured by PEFR, declines with increasing severity, underscoring the importance of timely assessment and intervention. While guideline-based management, including SABA, systemic corticosteroids, and supplemental oxygen, effectively stabilizes most patients, some severe cases did not receive optimal therapy, reflecting real-world practice variations in resource-limited settings. Early recognition, prompt treatment, patient education, and multidisciplinary care remain critical to optimize maternal and fetal outcomes in this high-risk population.
